# Responsibility for Funding Refractive Correction in Publicly Funded Health Care Systems: An Ethical Analysis

**DOI:** 10.1007/s10728-020-00423-9

**Published:** 2020-12-23

**Authors:** Joakim Färdow, Linus Broström, Mats Johansson

**Affiliations:** 1 Medical Ethics, Department of Clinical Sciences Lund, Lund University, 221 84 Lund, Sweden; 2 Department of Ophthalmology, Region Kronoberg, 351 85 Växjö, Sweden

**Keywords:** Health care needs, Publicly funded health care, Uncorrected refractive error, Visual impairment, Prioritization

## Abstract

Allocating on the basis of need is a distinguishing principle in publicly funded health care systems. Resources ought to be directed to patients, or the health program, where the need is considered greatest. In Sweden support of this principle can be found in health care legislation. Today however some domains of what appear to be health care needs are excluded from the responsibilities of the publicly funded health care system. Corrections of eye disorders known as refractive errors is one such domain. In this article the moral legitimacy of this exception is explored. Individuals with refractive errors need spectacles, contact lenses or refractive surgery to do all kinds of thing, including participating in everyday activities, managing certain jobs, and accomplishing various goals in life. The relief of correctable visual impairments fits well into the category of what we typically consider a health care need. The study of refractive errors does belong to the field of medical science, interventions to correct such errors can be performed by medical means, and the skills of registered health care professionals are required when it comes to correcting refractive error. As visual impairments caused by other conditions than refractive errors are treated and funded within the public health care system in Sweden this is an inconsistency that needs to be addressed.

## Introduction

In welfare states, society is responsible for ensuring the equitable provision of basic welfare of its citizens, and it discharges this responsibility in part through public health care [16]. Such health care systems allocate resources on the basis of *need*, the idea being that resources ought to be directed to patients, or the health program, where the need is considered greatest. Support for this principle can be found, for example, in declarations and legislation in Sweden and the UK—countries with large shares of publicly funded health care [8, 28].

Even within public health care significant health care needs are occasionally left unmet in individual cases. While this is unsatisfactory and ought to be addressed, it is perhaps only to be expected in complex health care systems with a large number of caregivers and a plethora of rules and regulations. The exclusion of entire domains of what appear to be genuine health care need from publicly funded health care altogether, on the other hand, is more problematic. For example, Sweden’s decision not to fund dental care (for adults) on the same conditions under which it funds other publicly funded health care has been criticized for being both arbitrary and unfair [1]. The funding of corrections for eye disorders known as refractive errors is another such domain, and the one that this article focuses on.

Refractive errors arise when deficiencies in the structure of the eye cause light rays to make uneven projections on the retina, resulting in cloudy, unfocused appearances of observed objects.^1^ They cannot be prevented, but they can be treated by refractive correction. This may involve spectacles, contact lenses or refractive surgical procedures. The general aim is to change the projection of the light rays so that sharp images of the observed images are restored. Corrections of refractive errors cannot be expected to be funded by public means, not even in predominantly tax-funded needs-based systems. In Sweden, for example, spectacles and contact lenses are generally not publicly funded means for alleviating such conditions for individuals over the age of 19 years [27]. Citizens above that age, with rare exceptions [30], have to pay for ophthalmic examinations and whatever is needed for refractive correction themselves. This situation has received surprisingly little attention considering the impact it has on many citizens.

The aim of this article is to explore the moral legitimacy of the current allocation of financial responsibility for refractive errors, assuming a needs-based prioritization framework along standard lines. Our main illustration will be the case of Sweden, but the analysis is significant also for other countries where the funding of refractive corrections is not considered to belong to the health care sector, such as in the UK and Norway. The analysis also generalizes beyond the issue of refractive correction, as the article will in effect illustrate how, more broadly, a complex medical condition which presents in various ways can create medical needs that are not always regarded as such by professionals and the individuals afflicted with them, and how the financial responsibility for meeting these health care needs may to a large extent be based on tradition rather than ethical analysis.

The article is structured as follows. In the next section we lay the ground by articulating why it makes sense to characterize a correctable visual impairment arising from refractive error as a *health care need*. While this is a natural point of departure, obviously, it does not settle the presumably more contentious issue whether refractive corrections should be part of publicly funded health care. For that, one thing that must also be established is the severity, or magnitude, of the need. We address this issue in the “Refractive Errors in a Prioritization Setting” section, where we argue that while the population in need of refractive corrections is anything but homogenous, for at least some of these individuals their condition could be quite severe and does constitute a considerable health care need, one which is at least equally deserving of public funding as the alleviation of conditions that are considered deserving of public funding based on the severity of the need. In the “The Case *Against* Public Funding of Refractive Corrections” section we turn to arguments that could be taken to support the current policy where refractive corrections are generally not funded with public means. The article ends, in the “Conclusion” section, with some concluding remarks.

## Are Correctable Visual Impairments Caused by a Refractive Error Health Care Needs?

Does poor visual acuity caused by a refractive error constitute a health care need? To many of us, an affirmative answer may seem straightforward. However, the way the slow degradation of visual acuity may appear to be a simple fact of life, and the circumstance that the most common remedy of the problem—buying glasses—doesn’t in any way involve health care, may for some introduce uncertainty. Also, while there may be little explicit opposition to the contention that poor visual acuity is a health care need, a comprehensive ethical analysis of the issue must arguably start here. Before addressing whether it is indeed a health care need, and the further question whether such a need would be one that publicly funded health care has a responsibility to try to meet, we shall however briefly comment on the even more fundamental issue: is it a *need* at all? To address this, consider first:

*Case 1: Paul*

Paul has a refractive error in both eyes affecting his visual acuity. The error impairs his ability to distinguish fine details at distance. Without spectacles Paul cannot read the subtitles clearly when watching TV. He has some difficulty following screen presentations at work, and it is more difficult for him to drive, especially at night. Occasionally Paul cannot recognize people he knows when he sees them at a distance, or read signs he is not close to, such as timetables at train stations. Wearing glasses, he can do all these things without any difficulty.

Does Paul *need* glasses (or some other means to correct his refractive error)? Intuitively he does, as spectacles will contribute to his visual acuity. This accords with common philosophical accounts of need (in health care prioritization settings) according to which needs are inherently *instrumental* [14]. According to such understandings of need, a person x needs something y in order to accomplish something z, where z is the “purpose” in relation to which y is instrumental [15]. Another way to frame the instrumental aspect of need is in terms of “condition-intervention pairings”, where a particular intervention is needed if it has some potential to provide benefits relative to a specific condition [12]. Thus, being in a poor state is not enough for constituting a need. If there is no intervention from which the individual can benefit, then there is no need.^2^

Now, Paul has a need for refractive correction not simply because he *wants* glasses, as he might have a desire for ice-cream or watching a movie. On the contrary, the glasses seem to be necessary for him to achieve ends that we can all agree are important, or valuable, not just to Paul, but to just about anyone who finds themselves in the same circumstances. Hence, Paul’s need arguably relates to an end which is valuable in a more objective, or at least interpersonal, sense. This, is in line with an influential approach to needs, according to which, roughly speaking, we need things without which we would be harmed [34]. A need, here, constitutes a state of dependency for the afflicted individual, a dependency in respect of not being harmed [4].

In the health care prioritization context, these and other aspects of needs have to be elaborated. Here we can draw on the work of Hasman and colleagues, who identify what they take to be three distinct interpretations of health care need [12], but that according to our view is better understood as three *dimensions* of need. According to the first of these, needs are understood in terms of the individual’s state of health before treatment. An intervention is then said to be needed if, and only if, the individual’s initial health state (the non-intervention health state) is *sufficiently poor*. This, of course, presupposes that there is some threshold above which the individual should not be considered to be in a poor state. Just where that threshold is located could be difficult to determine, but there is no shortage of examples of states that we could all agree are unacceptably poor. Life-threatening diseases, like cancer, and serious neurological conditions like multiple sclerosis may exemplify such poor conditions. Individuals being in such conditions will benefit from even the slightest improvement of their health status [12].

Applied to our example of individuals with refractive errors, the non-intervention health state would be the state which individuals are in prior to refractive correction. Does poor visual acuity due to refractive error qualify, on this interpretation? In this connection, let us also consider another example, Allison:

*Case 2: Allison*

Allison is 33 years and has a larger degree of refractive error than Paul, resulting in a more profound impairment of her visual acuity. The condition first appeared when Allison was at middle school (age 11) and her teacher reacted to Allison’s inability to see what was written on the panel in the classroom. Allison has worn spectacles ever since, except when she is reading books in bed at night. She has no problem reading or seeing things at near range, but everything at far range is blurry. The refractive power of Allison’s eyes altered, in its degree, quite often during the first few years, and her glasses had to be changed almost every six months. Now, however, her condition is more stable, although her glasses still need to be changed twice each year. Without any intent to downplay Paul’s need, Allison is arguably in a worse state than he is, and benefits even more from using spectacles. In addition to the difficulties encountered by Paul, Allison would not be able, or legally allowed, to drive a car. Hence professions demanding a driver’s license would be closed to her. Shopping at grocery stores and supermarkets would also be difficult, since she would have to get very close to signs, price tags and the products she would like to buy in order to get access to product information, recognize distinct things like different sorts of fruit, or judge the quality of meat, etc. With spectacles, Allison can do all of these things without difficulty.

Now, does it make sense to speak of Paul and Allison being in a poor condition? Given the specific demands that individuals face in modern society, and the associated low subjective quality of life which typically goes along with not being able to live such a life on an equal basis with others, surely it does. Without refractive correction, Paul and (even more so) Allison would face considerable difficulties in taking part in society—both when it comes to daily activities like buying food and traveling by public transport, and in social life (since they could not recognize people at a distance) and not least in the labor market. Many professions today require a driver’s license, mandating a certain level of visual acuity.

The second dimension of need relates to what has been termed *normal functioning range*. On this view an intervention is needed if it enables an individual to achieve a certain target—a particularly important state of health, as it were. The intervention is said to be needed if the individual is in a state below that pre-defined minimum health state where he or she is “sufficiently healthy”, before the treatment, and above that state after the treatment. It could be an intervention that is needed for an individual previously bedbound to be able to move around [12]. Hasman et al. describe normal function in terms of a state of health where the individual is sufficiently healthy and autonomous to participate fully in society. Paul and Allison, *if* they are provided with refractive corrections, will attain visual acuity at what we would consider a perfect level, so the relevant intervention would indeed bring them into the “normal functioning range”.

Finally, the *significant gain dimension* focuses on the magnitude of the benefit that the intervention will provide for the individual. Perhaps the most paradigmatic examples of such interventions are those that involve life-saving. But many other interventions offer significant gains to those intervened on. Refractive corrections do indeed have the potential to move individuals from states of severe functional dependency to states of excellent visual function. Without such corrections, individuals like Paul and Allison would encounter various difficulties in their lives, as described above, but with proper refractive corrections they will be able to take an active and autonomous part on the same terms as everybody else.

Having argued that Paul and Allison do need refractive correction, we now turn to the critical question whether their needs are *health care*, or *medical*, needs—i.e. the kinds of need that we could, or should, expect health care to try to meet (if its resources and prioritization principles allow). While Hasman and colleagues frame their analysis as one of health care needs, simply pointing to the fact that refractive errors can be described in terms of the three dimensions mentioned above is clearly not enough. After all, these dimensions are equally suited to other needs as well, ones which clearly do not belong to health care, such as food for the hungry, clothes for the freezing, or education for the uneducated. Hence the question remains whether the need for refractive correction concerns health care.

What counts as a health care need is not well defined. And we make no claim to be able to *prove* that it ought to be the responsibility of health care to consider providing refractive corrections to those in need of them. As we shall see, however, Paul’s and Allison’s needs for refractive correction exhibit quite a few features of paradigmatic health care needs, so at the very least, anyone who would deny that the provision of refractive corrections has anything to do with health care would owe us an explanation as to why.

First Paul’s and Allison’s medical conditions create needs that certainly appear to concern *health*. Health has been philosophically described in terms of individuals´ abilities of realizing their vital goals [20] and this way of describing health, though contested [7], corresponds with how the WHO defines health, namely as “a state of complete physical, mental and social well-being and not merely the absence of disease or infirmity” [35]. An individual’s ability to visually discriminate details, the subject of this article, is undoubtedly essential for human beings, their functioning in modern society, and their ability to realize their life plans. It arguably captures something important about *health issues*—namely, that it matters to what extent they negatively affect the everyday lives of the people suffering from them. Put differently, the medical states of Paul and Allison, prior to intervention, cause them to be unable to *do* certain things. Thus, they relate to some kind and degree of disability.

Second, Paul’s and Allison’s needs relate to conditions that are officially considered medical in nature. Conditions of, and treatment options for, refractive error are included in the curricula in medical schools, they are thoroughly described in medical textbooks and subjected to intensive research in medical fields like optometry and ophthalmology. Further, refractive errors do have medical diagnostic codes in the ICD-11-system provided by the World Health Organization [36]. Thus they are acknowledged medical conditions, and in that sense we are well within the medical domain.

Third, at one stage or another, the provision of refractive correction often calls for the special competence of health care professionals. Refractive services, i.e. diagnosing and characterizing refractive errors, require registered opticians or ophthalmologists. Trying out the correct refractive corrections (in terms of diopters and degrees of astigmatism) similarly requires specialist assistance. Further, surgical procedures to correct refractive errors, whether it be by laser treatment of the surface of the eye or invasive eye surgery, simply cannot be conducted without ophthalmic surgeons.

Finally, modern health care often provides aids and services which help the patient in ways that do not amount to medical treatment, and do not alter the underlying medical condition. The clearest case, in the Swedish system, is hearing aids, which, if hearing ability is below a predefined threshold, are publicly funded.^3^ Another example is the provision of wheelchairs. Actually, a recent Swedish governmental report on medical aids [29] defines medical aids in such a way that refractive corrections, at least at a certain degree of refractive error, do meet the standards set up for the proposed definition. The report proposes a definition of medical aids that is based on individuals’ ability to manage in daily life activities, to orientate themselves in unfamiliar surroundings, and to communicate with others, among other things. Further, the report recommends that medical aids should be provided within the publicly funded health care system.

To summarize, the need for refractive correction exhibits a number of characteristics of what we would in other contexts consider to be health care needs. While this does not settle the issue of whether, at the end of the day, it ought to be viewed this way, it does, at least, place the burden of argument on anyone who would object to this classification. That the need for refractive correction should be viewed as a health care need is, of course, consistent with the position that such correction should not be funded publicly. In a needs-based publicly funded system it is crucial to consider in addition the severity of the conditions under discussion, and the magnitude of the corresponding needs. This is, therefore, what we shall do now.

## Refractive Errors in a Prioritization Setting

The question whether refractive corrections ought to be publicly funded cannot be tackled without some idea of how to measure, or weigh, the needs of individuals seeking them. Society may decide that many of the needs we acknowledge as health care needs are simply not important enough, given scarce resources, to be eligible for public funding. How do refractive corrections fare in this regard? Making such assessments is challenging, of course, as the subject of discussion is an entire domain of conditions. The problems that arise range from the moderate to the significant. What role society *ought* to have in promoting the health and welfare of its citizens is a too basic issue to be dealt with in this article, as is the issue of how in general one ought to assess the severity of needs. For present purposes we will simply assume the core ideas in a tax-funded and needs-based health care system (such as the ones in Sweden, Norway and the UK), about how needs ought to be assessed, and focus on whether the choice not to fund refractive correction introduces problematic inconsistences in such systems.

There are at least two basic roles for the principle of need in processes of allocation and priority setting in health care [15]: the principle should help us determine both who has a justified claim to treatment and care, and how treatment and care should be allocated among those who have such claims. Agreeing on the formulation of such a principle is anything but easy, as Juth convincingly shows in his article. For present purposes, however, consensus over a general prioritization principle is not required. It will be sufficient to decide whether the need for correction among those with refractive error is significant, or serious, enough to merit public funding.

### Is the Need for Correction of a Refractive Error Significant Enough to Attract Public Funds?

How can we assess the needs of individuals with refractive errors in a standardized fashion that allows comparisons to be made with other health care needs? We need an assessment tool that allows for comparisons, and preferentially some kind of grading, between various health states.^4^ Such an assessment tool, the Severity Framework, has been developed by the Swedish National Center for Priority Setting [24]. This framework is devised to concord with the Swedish platform for priority setting [25], but there is nothing uniquely Swedish about it. On the contrary, it reflects the ambitions of a need-based health care system in that it allows various health conditions to be ranked and assessed together with relevant interventions in order to provide bases for prioritizing and resource allocation. In this respect, it mirrors considerations central to guidelines in Norway [2, 3] and the UK [19]. The way in which severity and magnitude is assessed also harmonizes with (although much less detailed) ICF-checklist for functioning and disability provided by WHO [38].

Central to the Severity Framework prioritization model is quality of life,^5^ covered by the following aspects: *impairment of bodily functions* (including physical and psychological impairment), *activity limitations* (practical consequences of ill health), *participation restrictions* (social consequences), and the *occurrence and duration* of these problems, plus the *risk of future ill health* [5]. The various aspects can be graded in a special matrix and given the following grades: very high, high, moderate, low and none.

Let us ask, then, what an assessment based on this model would conclude if the condition being assessed was one of severe visual impairment due to refractive error. To address this question, we can consider the following example:

*Case 3: Leonard*

Leonard is 32 years old and has a prominent refractive error resulting in a severe visual impairment. The condition first presented when Leonard was a toddler, and he began wearing spectacles at age two and a half. Since the age of 15 he has worn contact lenses permanently during the daytime. The refractive condition is unstable, and the power of his lenses has to be adjusted after almost every visit to his optician. He visits every six months. Leonard takes out his contact lenses at night and keeps spectacles on his bedside table so he can find his way to the bathroom and deal with other things that may happen at night. At very close range, immediately in front of his eyes, Leonard can see sharply. Beyond that, all is a blur without correction. Leonard would experience various difficulties without his contact lenses and spectacles. Already at home, most chores would be very demanding: chores like cooking, doing the laundry and taking care of children, to mention just some. Shopping at the grocery store would be impossible without help. Getting around in town would be potentially dangerous for him, as he would not be able to identify obstacles in his way or see moving vehicles like cars or bicycles. Walking on uneven roads or off-road, let alone on fresh snow or ice, would be very demanding for Leonard. And communicating with other people would be a struggle, given Leonard’s inability to read facial expressions. Traveling by bus or train would be impossible without the help of someone else. Without considerable adaptations to his working environment, it would also be impossible for him to uphold a professional job, and most positions on the labor market would be closed to him. When wearing his contact lenses Leonard’s visual function is flawless. The annual cost for contact lenses is at least €400. To that should be added the cost of Leonard’s eye examinations (€10–20) and extra spectacles (€200 minimum).

An assessment using the framework provided by the Swedish National Center for Priority Setting would give the following result for Leonard’s condition. *Impairment of bodily functions:* Leonard cannot orientate himself in unfamiliar surroundings due to his bad vision, thus his bodily functions are impaired to a very high degree. *Activity limitations:* he will find considerable difficulties when it comes to shopping, doing chores at home and upholding a profession, thus the activities that are possible for him to do are limited to a very high degree. *Participation restrictions:* Leonard´s ability to socially participate is restricted to a high degree, not only due to difficulties when it comes to getting in physical contact with other people but also due to difficulties reading facial expressions. *Occurrence and duration:* Leonard´s condition would be shown to be constant and permanent. *Risk of future ill health*: there is no risk of future ill health due to his condition. Taken all together, the assessment shows that Leonard indeed is afflicted by a serious impairment of quality of life, in this framework. When it comes to the intervention part, we know that refractive corrections have the *potential* to move an individual like Leonard from a state of severe visual impairment to a state of perfect visual acuity. Thus, the patient benefit, in this case, would be quite considerable.

But even individuals with more moderate impairments of visual acuity, such as Paul and Allison, have significant needs on this kind of assessment model. Modern societies make certain demands of their inhabitants’ abilities to distinguish fine details. The demand for certain levels of visual acuity will be noticeable both on the labor market, in social life, and in leisure activities. Some of these demands have been mentioned above, and they can be seen in the short description of the challenges Paul and Allison have to face without spectacles. Without refractive correction, Paul’s and Allison’s abilities to perform an active and autonomous role in society is diminished. In the absence of that correction, Paul and Allison are therefore harmed. This gives the character of their needs a certain moral weight.

### Is Today’s Practice in Conflict with the Principle of Equality

We have so far considered the need for refractive correction in absolute terms, relying on the notion of quality of life. Another approach, however, is to compare the corrective intervention with cases that are *already* funded within the health system—e.g. other states affecting vision in which the relevant interventions aim to improve an individual’s visual acuity. Should the two kinds of cases be very similar, in terms of the needs they contain and the interventions adopted to meet those needs, the case for funding refractive correction would be even stronger, as like cases should presumably be treated alike. Let us consider Harry, an individual with cataract:

*Case 4: Harry*

Harry is 68 years old and suffers from cataract in both of his eyes. His visual function at distance has deteriorated gradually over the past few decades, and it is impaired to such a degree that he has considerable difficulty finding his way about in unfamiliar surroundings. If he is going somewhere, he has to be helped and led by someone else. The advised intervention for Harry’s condition is cataract surgery. Cataract surgery is very effective in most cases, and with it, severe complications are rare [31]. Intraocular lenses can be implanted, and surgery can be performed on both eyes on the same day. Postoperatively, Harry’s distance vision would be expected to be excellent. He would certainly be able to work outdoors again, and to be able also to ride a bike or drive motor vehicles. He will need to use reading glasses to see sharply at close range. In the Swedish system, Harry will only have to pay a small fee for the preoperative visit, the date of surgery and the postoperative visit one week later (totally approximately € 90). The remaining costs (€ 900–1000) are covered by public funds.

Harry’s case highlights a possible inconsistency in the publicly funded health system. His condition is one that is treated within the publicly funded health care system in Sweden, and the same allocation of financial responsibility can be seen in connection with many other conditions causing visual impairment.^6^ Now, Leonard and Harry suffer from visual impairment of the same magnitude, and in both cases interventions with the primary aim of visual improvement are warranted. Furthermore, in both cases significant improvements to visual acuity are expected if the interventions are performed. In Leonard’s case, refractive correction is appropriate. The options are: spectacles, contact lenses or surgery of some sort—either laser technology changing the refractive power of the cornea or replacement of the original lens with an artificial intraocular lens. And if Leonard opts for such a lens removal procedure he will have to pay approximately €2500 per eye in the private sector. In the case of Harry, cataract surgery is appropriate. This involves the removal of the opaque original lens (damaged by cataract) and its replacement with an artificial intraocular lens—essentially the same procedure as the one we mentioned last when going through Leonard’s case. In Harry’s case, but *not* Leonard’s, the surgery is offered within the publicly funded health care system. One can argue that if the intervention on Harry is deferred, further optical deterioration may in time result, and that in some instances of cataract ocular inflammation and elevated intraocular pressure requiring special treatment occur. However, this is not relevant in Harry’s situation, where the primary aim of surgery, at least in his current situation, is improvement of vision.

If indeed there is an inconsistency here, it highlights an arbitrariness in the way the health care system works. And the problem is one of fairness—why help one person but not another, everything else being equal? There are three strategies by which to avoid or deal with this inconsistency. First, one can change the current health funding arrangements and start paying for refractive corrections for people in situations like Leonard’s, with public money. We take this to be a serious option. Second, there is the option of withdrawing public funding for treatments like the one Harry will be offered, implying that this was the wrong thing to do to begin with. Few are likely to accept this alternative. Third, one can try to argue that there is no real inconsistency—that there are good reasons for the funding difference. We shall now look at this third option—i.e. at possible arguments in favor of the current arrangements.

## The Case *Against* Public Funding of Refractive Corrections

As we have illustrated, individuals with refractive errors need refractive correction if they are to do various things, including performing everyday activities, managing certain jobs, and accomplishing various goals in life. Moreover, these needs, as we have seen, often fit well with how health care needs are typically understood. We have also argued that those who suffer from refractive error seem to have a legitimate claim to treatment, since their needs (in some cases, at least) are significant, and without treatment they would be in a quite severe state of impairment. Is there, however, a case to be made for nonetheless preserving the status quo? Whether a certain funding policy should be kept in place, after all, perhaps ought to depend on broader considerations than the ones so far addressed.

### Risk of Stigma and Medicalization

Certainly lightweight as a free-standing consideration, it is still worth noticing that changes to the current arrangements, so that refractive corrections are no longer financed and provided privately, but are brought into the public health system, could stigmatize some individuals with refractive error. Stigmatization works by distinguishing a certain group, linking this group to negative stereotypes, and thereby ensuring it suffers from discrimination and loss of status [10, 23]. Spectacles—the most common method of correcting refractive error—are very obvious markers, the mechanism of the stigmatizing process being straightforward. Today, however, the wearing of spectacles is not obviously linked to negative stereotypes; some individuals might even choose to wear spectacles without refractive power for fashion reasons. If the provision of spectacles were moved into a publicly run dispensing system, conditions could however change, and risks of stigmatization might appear, especially if the publicly funded spectacles were easily recognizable as such, e.g. from the limited assortment of characteristically simple frames—a reality that existed in the NHS between 1946 and 1986 [11]. Mechanisms of stigmatization could work through links, made by other individuals, between individuals wearing the funded spectacles and negative stereotypes, such as those of disability and/or dependency on financial support or some other kind of support.

Further, medicalization could amplify these negative conceptions by connecting the individuals with refractive error more closely with notions such as illness and impairment. The risk of causing harm would be even more apparent if the system was organized so that only individuals with the most serious need for refractive correction were eligible for the publicly funded services. If the system were to be organized in that way, and only individuals like Leonard were entitled to publicly financed refractive services, extra caution would need to be taken in order not to stigmatize this group further—bearing in mind the extent of the disability that these individuals already is afflicted by, and their absolute need for refractive correction.

If contact lenses were included in the publicly funded service (costs for contact lenses and glasses are roughly equal), some risks of stigma could be minimized, at least for those choosing contact lenses. But individuals would still need to visit health care facilities for examinations, refraction services and checkups. There would also be a risk of self-inflicted stigma, in the sense of viewing oneself as an individual who is dependent on financial support.

One measure to mitigate the stigmatization of individuals with refractive error is public funding for refractive surgery. Today such surgery has the potential to make spectacles or contact lenses unnecessary for most individuals with refractive error, and thus further contact with health care centers would not be needed. The cost of incorporating these surgical procedures in the publicly funded health care system would be considerable, however.

Another way of mitigating stigma would be to use systems based on vouchers, or subsidies, based on individuals’ needs for refractive correction. A voucher system, or a subsidy system like the one in Sweden (currently targeting children and young persons), could be constructed and be operated side-by-side with the private provision of refractive corrections. Through these measures, the process of stigmatization would likely be prevented.

### Freedom of Choice

One thing that could be seen as working satisfactorily at present is the way the current system allows for individual choice. Today the commercial spectacle-dispensing market offers good accessibility, supply and an assortment of glasses of different materials, grinding-designs and designs of frame (i.e. qualities that are aesthetic and concerned with comfort, and not directly connected with refractive correction). Contact lenses exist as an option for individuals with special requirements—primarily, aesthetic requirements, or requirements connected with certain activities such as outdoor work or sport. The range of contact lenses on offer today is very good. There are contact lenses with various properties, even for the correction for astigmatism and presbyopia. For those who want a permanent solution for their refractive error or cannot tolerate contact lenses (e.g. because they have ocular allergies), the commercial market offers a wide variety of refractive surgical procedures. In these conditions, customers have genuine freedom of choice across a wide range of, as it were, selling points (many of which are not directly related to refractive correction). It is by no means obvious that this freedom is transferable to the public sector—at least, in the absence of a hybrid model where patients are allowed to part-fund products that are not available within the public sector [9]. Now, nothing rules out a parallel, private system like the one we have today. Systems based on vouchers, or subsidies, for individuals with the need for refractive correction could be constructed, and then provision could remain in the hands of private opticians. Freedom of choice may then remain. Those who value the freedom to choose can turn to the private market.

### Precautionary Thinking: Don’t Meddle if it’s Working!

One possible strategy to defend the current arrangements is to call for precaution. If the system works in the sense that relevant needs are satisfied in it (to a sufficient degree), we should hesitate before making changes, as this might lead to negative, unintended side-effects. For example, there might be a risk of introducing additional costs and a risk of displacement of resources. Moving financial responsibility for refractive corrections for all, or some groups of, individuals with refractive error into the public sector may, for instance, require additional funding and/or the reallocation of funds currently devoted to other health care activities (this would certainly be the case if every sort of refractive correction, including refractive surgical procedures, was moved into the publicly funded health care system). Such re-prioritizing could mean that some health care measures that are publicly funded today have to be de-prioritized and perhaps not even funded anymore. It may even turn out that the new system would not be able to manage the burden of treating a large number of new patients, and this could lead to the breakdown of a system that presently works quite well.^7^

The current funding policy could well be based more on tradition than ethical analysis. But today’s citizens have adapted and become accustomed to these funding arrangements—to the fact that spectacles, contact lenses and other forms of refractive correction must be financed by the afflicted individuals themselves. This adaptation may not only contribute to explaining the near absence of critical discussion of the current policy but actually give us a reason to embrace a conservative approach when it comes to proposing changes to the financing and provision of refractive corrections. Through the process of adaptation, people may over time have to endure fewer of the costs of an unfair policy, and radically changing a well-entrenched policy may lead to hard to predict challenges in adjusting to a new order, with the possibility of frustrating important interests, at least short term. Maybe, in other words, one shouldn’t meddle with a well-established policy if it’s “working”. This obviously goes hand in hand with *market acceptance*, which here includes the existence of sufficient incentives for commercial spectacle-dispensing services, including opportunities for entrepreneurs and investors to invest money and make profits. Without such market acceptance, we would not expect the market to be able to manage the situation. Clearly, market acceptance requires spectacles and contact lenses to be affordable to most people (even refractive surgery has found its market, with entrepreneurs presently offering it in all major cities in Sweden). It also depends on people’s willingness to pay, on a large number of individuals being in need of some kind of refractive correction, and on the fact that the corrections aims at improvement of quality of life.

Relatedly, it may be suggested that products which are frequently found in people’s homes and available on the high street should not be prescribed as medical aids.^8^ The publicly funded health care system in the US shows some similarity to this view. When Medicare became law in 1965, it was stated that items that were “routinely needed and low in cost”—hearing aids and vision aids were mentioned as examples—should not be included [33]. What counts as “low in cost” is debatable, of course, but the Leonard case highlights that costs, or rather affordability, might vary considerably for citizens, and arguably be significant for some. At the same time, the affordability argument does sometimes seem to find its target. Consider, for example, the case Gretchen:

*Case 5: Gretchen*

Gretchen is 54 years old and suffers from presbyopia, a common condition affecting most individuals in the later stages of middle-age. The condition results in blurred vision at near range, and Gretchen began to be aware of the issue at the age of 47 when she encountered difficulties reading and with tasks at the computer. Following advice from her GP, Gretchen bought reading glasses at the supermarket where she shops (€ 10–20 a pair). She has since replaced the glasses to raise their refractive power twice, and now wears a pair with the power of +2.5 diopters. She tries them out herself in the shop. Gretchen wears the glasses when she reads, when working at the computer, and for looking at photos and other things at near range. She has no difficult focusing on things at a distance.

Gretchen could be viewed as someone at the lower end of the needs scale when it comes to refractive error, although one should not confuse the ease with which she overcomes her problem (and the low price of the spectacles) with the magnitude of her need. Her need is certainly not immaterial. Without reading glasses Gretchen would not be able to perform any tasks demanding visual acuity at near range, such as reading, writing, typing or other computer work, looking at pictures, doing her make-up, cutting her grandchildren’s toenails. Still, *in her case* there might be a reason not to incorporate refractive correction interventions in the publicly funded health care package. Having persons in this situation to solve the problem themselves might be much more effective than trying to manage it via the public health care system. Indeed, her solution would certainly be cheaper for her than paying the general fee for a health care visit to get a prescription for reading glasses. In short, the reasoning we are considering—that society should not fund what is readily available at relatively low cost—makes sense in some cases but not others. Unsurprisingly, we are dealing with a heterogenous population when it comes to needs.

But would not the sheer cost of providing refractive corrections within the public health care system be an argument in favor of maintaining the current arrangements? In the UK, dentistry and spectacles were initially included in the NHS and provided free of charge. User charges were introduced at a later state, when NHS expenditure had grossly exceeded estimates [32]. Spectacles were removed from the public health system at that point mainly for fiscal reasons.^9^ This straightforward argument hardly convinces anyone—at least, not without linking it to an assessment of public need and the alternatives. If the mere fact that something is expensive were a satisfactory argument, we could shut down the publicly funded health care system altogether. Also, there is certainly a downside to outsourcing to the free market, as opticians in such a system are both health care professionals and sales people. And there are clearly economic incentives to sell products of (apparent) higher quality and price. A change to a publicly financed system would alter these conditions.

### Democratic Legitimacy

There seems to be a broad acceptance of the current situation of funding refractive corrections in Sweden. This is an acceptance that seems to go for most other countries as well. Although there has been no national referendum on whether refractive corrections should be funded with public money in Sweden, one could certainly argue that the current state of affairs is nonetheless politically sanctioned in an open and democratic society. Further, as already indicated, there is little public opinion against the current situation, and this might be taken as a sign of a system that is working well.^10^ At any rate the status quo seems to be broadly accepted. On the other hand, there is arguably more to democracy than the mere tolerance of the majority. Most importantly, protecting the rights and interests of vulnerable groups and minorities is typically regarded to be a central part in the democratic welfare state. In other words, while there is an obvious sense in which the current order has democratic legitimacy, this sense is limited. The policy could be viewed as giving insufficient consideration to the perspectives of those who are the most negatively affected by it but may not realize that principles of fairness could put it into question.

## Conclusion

As with any other prioritization issue, the issue of financing refractive corrections introduces tensions between various interests. The moral justification of the current exclusion-policy comes down to how these interests should be weighed, and the probability with which they are in danger of being frustrated. On the one hand, there are reasons for keeping the current policy; values connected to avoiding stigmatization of individuals with refractive errors, urges to preserve freedom of choice for this group, avoiding additional costs and reallocations in public health care systems and a general call for precaution of a system that in certain ways seems to work relatively well. On the other hand, refractive errors do give rise to health care needs, in some cases quite considerable health care needs.

The fact that refractive errors give rise to significant needs, we have argued, provides public health care with a weighty reason to fund correction. Today, with rare exceptions, the provision and funding of corrections for refractive error is not considered the responsibility of the public health system in Sweden. Optical examinations, and any ensuing refractive correction, are hived off to the private sector. Quite why this is so is, as we have shown, not self-evident. The relief of correctable visual impairments fits well into the category of what we typically consider a health care need. The study of refractive errors does belong to the field of medical science, interventions to correct such errors can be performed by medical means, and the skills of registered health care professionals are required when it comes to correcting refractive error. That refractive error creates a real need has been shown here. Individuals with refractive errors need spectacles, contact lenses or surgery to do all kinds of thing, including participating in everyday activities, managing certain jobs, and accomplishing various goals in life. We thus contend that, where the individuals’ needs appear to be equivalent, the burden of proof remains with those who argue that some interventions, but not others, should be funded publicly. Under any circumstances, this kind of inconsistency is one that needs to be addressed.

## Footnotes

1 Refractive errors are classified as myopia, hyperopia, astigmatism and presbyopia or combinations of these aberrations. Uncorrected refractive errors are the most common cause (43%) of visual impairment globally [13, 22] and constitute a major health issue in many developing countries [37].

2 This is important for the explorations in this article since many individuals with poor visual acuity are affected by ophthalmological conditions where no sight-improving interventions are available. Atrophic macular degeneration, lesions of the optic nerve and CNS-lesions are some such examples.

3 The same goes for low vision aids. However, only, if the best corrected visual function is below a certain level (8).

4 There are several assessment tools for assessments of quality of life in various conditions, including conditions of visual impairment. Some of these tools have been used in studies focusing ophthalmological conditions [6, 17, 21]. The study by Park et al. [21] was indeed designed for comparison between health care states affected by visual impairments and health states affected by other causes, but it excludes visual impairments solely due to refractive errors. This may be because it is quite commonly assumed that the correction of refractive error is not a genuine medical intervention.

5 This is in keeping with the official position in Sweden. The governmental bill [26] based on the final report from the Swedish Parliamentary Priorities Commission [25] stresses the importance of assessing health care needs in the terms of the severity of a disease and its impact on quality of life.

6 Another example is edematous macular degeneration: here the treatments, including the necessary injections of pharmacological substances reducing the swelling on the retina, are paid for with public money.

7 It may have a negative effect on job opportunities for relevant professionals (opticians, their assistants, manufacturers of optical aids, et al.). However, this is too weak an argument to be taken seriously, as it seemingly implies that the market would be more ineffective in terms of how many ought to be employed, or that we should embrace a system simply because it creates such jobs.

8 Sweden’s position does not seem entirely consistent. The government report on medical aids mentioned above [29] proposes a definition of medical aids that means refractive corrections (at least, in states of advanced refractive error) do qualify as medical aids.

9 Currently, a subsidy system consisting of free eye tests and optical vouchers that are available to certain groups exists in the NHS [18].

10 On the other hand, politicians in the Swedish parliament did send a signal, through the law passed in 2015 prescribing that spectacles and eye examinations should be funded (or at least subsidized) for individuals under the age of 19 years, indicating that refractive corrections should be financed for at least one group of individuals [27]. It should also be noted that there is nothing in the Swedish legislation, or government regulations, *prohibiting* health care interventions aiming at refractive errors from being performed in the publicly funded health care sector. Thus, there does not seem to be any legal hindrance for hospital administrators and heads of department to start treating individuals with refractive error, or more accurately prioritizing them, in just the way that they prioritize individuals with other states of visual impairment, i.e. according to need.
